# Note on Sulphate of Beeberine

**Published:** 1852-05

**Authors:** Henry S. Patterson

**Affiliations:** Professor of Materia Medica in Pennsylvania Medical College


*Note on Sulphate of Bebeerine.* By Henry S. Patterson, M. D., Professor of Materia Medica in Pennsylvania Medical College.

At a time when the discovery of a substitute for Sulphate of Quinia is a topic of general discussion, it may not be inappropriate to call the attention of the profession to a substance, heretofore noticed, but too generally neglected. The Sulphate of Bebeerine has been shown, by Dr. Maclagan, of Edinburgh, to be a medicine of very considerable anti-periodic power, closely resembling the corresponding salt of Quinia, and in many respects equal to it,—possibly superior. It is obtained from the Bebeeru or Green-heart (*Nectandra Rodiei*) of British Guiana, a tree of considerable size and extremely abundant. The bark yields the alkaloid largely, but it is particularly abundant in the nut. A decoction of the latter is the ordinary popular remedy for intermittent fever in Demarara, and, as I am informed by an intelligent gentleman of that place, seldom, if ever, fails to arrest the disease. The nut may be collected in almost indefinite quantities, and could be obtained here, if a demand were created, for little more than the expense of collection and transportation. The process for separating the alkaloid is almost identical with that for quinia, and not more expensive. If, therefore, it proves on trial equal in efficacy to that alkaloid, we will have a cheap and effective substitute within the reach of all. The subject certainly deserves a more extended investigation than it has hitherto received. The object of the present communication is to invite attention to it, and induce the profession, in miasmatic districts, to give the remedy a fair trial.

Sulphate of Bebeerine occurs in shining brown plates, (sometimes with a greenish tinge,) is inodorous, and has a bitter, harsh, somewhat astringent taste. Like the Sulphate of Quinia, it requires an excess of acid for its perfect solution. It may be given in pill, solution, or powder. That it is a good general tonic, in small doses, is very evident. In the full anti-periodic dose it is more apt to disturb the stomach than the same quantity of Sulphate of Quinia, and occasionally vomits; but it possesses the advantage of being much less stimulating, and does not affect the head as that salt does. Dr. Maclagan asserts that it is "not so liable to excite the circulation or affect the nervous system," and Dr. Neligan adds, that "this conclusion is fully borne out by his experience." The patients who have used it under my care expressly state that it did not occasion in them the same headache and vertigo as the quinia had previously done. Its dose is stated at gr. i.—v., three or four times in the day. Neligan directs it made into pill with conserve of roses, or in solution with the addition of a few drops of Acid. Sulph. Arom. The anti-periodic dose may be stated at gr. xv.—xx.

A letter from my friend and former pupil, Dr. H. J. Richards, of Grey Town, Nicaragua, of the date of March 25th, 1852, contains the following: "I have used the Bebeerine, as you suggested, with uniform success in quotidian intermittents. I have since had no opportunity to prescribe it in remittents. All the intermittents of this coast, however, are comparatively easily treated at this season, and yield readily to both quinine and arsenic. The remittents and even intermittents of the fall months, are more virulent and often speedily fatal." Those months will certainly furnish a fairer test of Bebeerine; but it is something to know that, under existing circumstances, it produces the same effect as the Quinia.

Dr. Watt of Demarara thinks that it is tardier in its effects than the Quinia, not interrupting the paroxysms so immediately, but he also thinks that its effects are more permanent. The cases in which I have had an opportunity of using it, seem to confirm the latter opinion.

1st. A gentleman residing in Blockley township consulted me in September last concerning an obstinate and constantly recurring tertian intermittent, under which he had labored for a length of time. He stated that the Quinia always interrupted the disease, but that it inevitably recurred in two or four weeks. I gave him Sulph. Bebeer. ʒss. dissolved in ℥viij. water, a tablespoonful to be taken every four hours during the apyrexia. The next paroxysm was prevented, and he has had no return of the disease up to the present time (April).

2d. A. J. applied to me in October last, with a very similar statement. While residing in New Jersey, about six years since, he had a violent and protracted "bilious fever," since which time he has had, every month or two, an attack of "intermittent fever," which has been generally speedily arrested by quinine. Such was his account of the case. I found his tongue furred, his eyes icterode, his breath offensive, his urine scanty and high colored. The anorexia was complete and thirst considerable. He had a daily slight chilliness, followed by considerable fever and a slight sweat. I gave him a mercurial purge and on the next day fifteen grains of the Sulphate of Bebeerine. He complained of some nausea, but no disturbance of the head. The same quantity of Bebeerine was given on the two succeeding days, when, the paroxysms no longer recurring, it was discontinued. He remains free up to this period (April), and says that he enjoys better health than he has done for years.

If the permanent character of effect, which these cases seem to indicate, should be established by a more extended experience, we will have in the Bebeerine an agent of very great value, adapted to cases which have hitherto seemed uncontrollable, except by arsenic, to which there are so many objections. It is also much more speedy in its effects than the arsenic. Bouchardat (Ann. de Therap.) expresses his surprise that the Bebeerine has been so entirely neglected in France, where trial is daily made in agues with substances of inferior efficacy. I trust that the same remark may not long be made with regard to the American profession, but that the precise value of the medicine may soon be established by an adequate extent of observation.

